# Well-being of artisanal fishing communities and children’s engagement in fisheries amidst the COVID-19 pandemic: a case in Aklan, Philippines

**DOI:** 10.1057/s41599-023-01716-9

**Published:** 2023-05-12

**Authors:** Ronald J. Maliao, Pepito R. Fernandez, Rodelio F. Subade

**Affiliations:** 1 Department of Ecology, Pál Juhász-Nagy Doctoral School of Biology and Environmental Sciences, Faculty of Science and Technology, University of Debrecen, 4032 Debrecen, Hungary; 2 Aklan Research Center for Coastal Studies (ARCCS), Aklan State University in New Washington, 5016 New Washington, Aklan, Philippines; 3 Division of Social Sciences, College of Arts and Sciences, University of the Philippines Visayas, 5023 Miagao, Iloilo City, Philippines; 4 Division of Professional Education, College of Arts and Sciences, University of the Philippines Visayas, 5000 Iloilo City, Philippines

**Keywords:** Development studies, Education, Social policy, Economics, Health humanities

## Abstract

This study describes and explains the multifaceted effects of the COVID-19 pandemic on the socio-economic and psychosocial well-being of the artisanal fishing communities in Central Philippines. The state of child labour and their education amidst the COVID-19 lockdown were also explored. Four hundred artisanal fishing households, with 792 children, from the 10 coastal municipalities in Aklan province were surveyed in May–December 2020 through face-to-face household interviews. The COVID-19 pandemic worsened poverty in these highly vulnerable fishing communities primarily through severe disruptions in their fishing and marine tourism-related livelihoods. The proportion of households living below the Philippine poverty threshold of PHP12,030 (USD232.7) monthly for a family of five members increased from 78% pre-COVID to 91% peri-COVID. This economic impoverishment was more pronounced in larger families with limited income, as in the survey sites, where 41% of the households have more than five family members. Furthermore, 57% of the surveyed households believed that learning difficulty increased by 81% among children due to the blended online education modality. Amidst increased impoverishment, child labour intensified, and children stopped schooling. A significant decline in happiness index peri-COVID was also observed in the study sites indicating extreme socio-economic challenges. Contrary to expectations, however, interpersonal relations in most households improved, underscoring women’s stabilising and nurturing role. This latter phenomenon signifies that cooperative and nurturing actor relationships can be generated even in a crisis. Policies that mainstreamed local communities’ reproductive health, family planning, and programmes that diversify socio-economic, environmental, and technological assets must be renewed and promoted. The goal is to holistically improve human well-being by increasing or sustaining stocks of these assets to promote resilience and sustainability amidst crisis and complexity.

## Introduction

The COVID-19 pandemic resulted in a massive global economic downturn at its height in 2020. During this time, the global economy shrank by 3.36% (World Bank, 2022a), the global unemployment rate increased to 6.5% (United Nations Statistics Division, 2022), and the number of people living in extreme poverty worldwide rose to 9.4% (World Bank, 2022b). Prolonged home quarantine to contain COVID-19 infections were often associated with increasing domestic violence (Boserup et al., 2020), worsening gender-based abuses and children maltreatment (U.N. Women, 2020; Lawson et al., 2020), and deteriorating state of child labour worldwide (ILO, 2020). Indeed, the COVID-19 pandemic has become a global economic and medical catastrophe. It is also a psychological crisis causing people’s subjective well-being^1^ to decline (Zacher and Rudolph, 2021; Wang et al., 2020).

Classified as a natural hazard by the International Federation of Red Cross and Red Crescent Societies (Seddighi, 2020), pandemics like COVID-19 have been documented and studied, providing vital inputs to design interventions and appropriate policies by governments and partner institutions. This paper aims to contribute to this growing literature and focuses on the socio-economic and psychosocial well-being of artisanal fishing households in the Philippines. These issues are crucial to the resilience and sustainability of vulnerable communities, particularly during a crisis. Community resilience is the ability to utilise space or time and adjust to a disturbance. It is an adaptive response to potentially disruptive change that seeks to limit damage or seize opportunities for improving socio-ecological conditions (Harley and Clark, 2020). On the other hand, sustainability is a state in which society does not undermine natural and social systems (Fernandez, 2018). Community resilience during pandemics is affected by the interplay of many factors, such as institutional (e.g., government support), social (e.g., mutual support and protection), economic (e.g., resource availability), infrastructure (sufficiency of services), and demographic (e.g., psychosocial well-being) (see Suleimany et al., 2022).

Southeast Asian countries have been hit hard by the pandemic, which quickly spread across the region, upending local economies, livelihoods, and the general well-being of the people (Ferrer et al., 2021). The Philippines recorded its first COVID-19 incident in the first quarter of 2020, and as of November 2022, it ballooned to 4 million confirmed cases, resulting in 64,524 deaths (WHO, 2022). In response to the spreading pandemic, the Philippine government implemented one of the world’s longest and strictest lockdowns and quarantine measures by the first quarter of 2020 (Hapal, 2021). The pandemic-induced lockdowns restricted mobility and economic activities, substantially disrupting the supply- and demand-side economy and causing the displacement of labour supply. Key tourism-related economic indicators recorded particularly severe declines in output, with the transport and storage sector recording a 30.9% decline, while accommodation and food services output slumped by 45.4% in 2020 (Biswas, 2021). The real gross domestic product (GDP) of the Philippines in 2020 exhibited a 9.6% year-on-year contraction, the sharpest decline since 1946 (de Lara-Tuprio et al., 2022). The unemployment rate also doubled in 2020 to 10.4% from 5.1% in 2019 (Royandoyan, 2022). Consequently, the Philippine population poverty incidence rose to 18.1% (19.9 million Filipinos) by 2021 amidst the COVID-19 pandemic, exhibiting a countrywide average increase of 1.4% from its 16.7% (17.6 million Filipinos) benchmark in 2018 (PSA, 2022).

The COVID-19 pandemic affected the whole value chain of capture and culture fisheries (Bennett et al., 2020). These effects are more evident in artisanal fisheries (Ferrer et al., 2021), with low job multiplicity (Maliao et al., 2009) and limited access to traditional financial services and protection (Pomeroy et al., 2020). Indeed, six out of 10 Filipinos did not have financial savings (Taruc, 2015), and this trend was much worst in fishing communities (see Pomeroy et al., 2020). Due to mobility restrictions, the national and local lockdowns in the Philippines constrained fishers from going to sea and selling their catch. Fishers faced combined stress from lost income, inability to support families, shortage and increasing prices of essential commodities, and exclusion from government relief schemes (Bennett et al., 2020). Also, there was an expression of acute anxiety in not knowing how long the pandemic would last and whether they could return to their livelihoods. The pandemic-fueled fishing restrictions and China’s expansion into contested waters in the West Philippines Sea have worsened things for Filipino fishers (Santos, 2021a). Moreover, decreased human observer coverage and lapses in monitoring and enforcement peri-COVID led to increased illegal, unreported, and unregulated (IUU) fishing and incursions into areas used by small-scale fishers (Bennett et al., 2020; Ocampo, 2020).

Amidst the multifaceted COVID-19-induced economic and social perturbations is the risk of worsening the state of child labour in the fisheries sector. Vulnerable fishing communities with insufficient socio-economic protection systems scramble to cope with unemployment and diminishing household income. Child labour (i.e., those who are <18 years old) is work that impairs children’s well-being or hinders their education, development, and future livelihoods (FAO and ILO, 2013). The nationally envisioned Philippine Program Against Child Labor Strategic Framework for 2017–2022, in conjunction with the Sustainable Development 2030 Agenda under Target 8.7, targeted to end child labour. However, child labour in the Philippines remains present and alarming (PSA, 2021). Artisanal fisheries, including aquaculture, are less structured and are primarily characterised as an informal economy; hence, child labour is widespread and often unregulated in these sectors. Children often engage in capture fishing, aquaculture activities, and other peripheral works such as processing, marketing, gleaning, and unpaid training engagements. These activities often require long hours and therefore interfere with their schooling, exposing them to hazardous conditions in some situations. Although child labour is attributable to many factors, poverty is considered one of the principal causes of child labour. This trend is increasing amidst the economic shock during the pandemic (Calleja, 2020).

The issue of child labour was made worse when academic institutions were closed during the COVID-19 pandemic (ILO, 2020), affecting more than 1.2 billion learners globally and 28 million in the Philippines (UNESCO, 2020). Education delivery in the Philippines has shifted from face-to-face to home-based during school closures, adopting new blended learning modalities comprising online classes and offline modules (Tria, 2020). While the blended learning modalities provide flexible and inclusive access to education during the pandemic, this has exacerbated inequalities and difficulties between and among learners. In the Philippines, these challenges are often associated with unequal access to online education resources and asymmetrical readiness of learners and home-based mentors under the prevailing educational reform.

While the effects of the COVID-19 pandemic have permeated all facets of human society, they have disproportionately impacted negatively the socially disadvantaged and economically marginalised groups worldwide (see McNeely et al., 2020). Hence, it is crucial to understand how the most recent pandemic affects the resilience of artisanal fishing communities, often considered as one of the poorest sectors in Asia, particularly in the Philippines (Pomeroy, 2012). First, we investigated the impact of COVID-19 policies on household economic well-being in artisanal fishing communities in Central Philippines. The goal is to understand the repercussions of the COVID-19 pandemic on overall household economic resilience and explore their livelihood coping mechanisms. Second, we looked at the state of child (<18 years old) labour in the affected artisanal fishing households and the children’s performance under the blended online education modality. In times of crisis, child labour becomes a coping mechanism for many marginalised families (ILO, 2020). In addition, children’s educational performance under the new educational reform reflects an essential aspect of human well-being. Finally, we investigated the state of household-level demographic resilience using subjective psychosocial well-being (i.e., happiness index and household interpersonal relations) amidst the COVID-19 upheaval. During the height of the COVID-19 pandemic in 2020, nine out of every 10 Filipinos were under severe stress, eroding their overall life satisfaction (SWS, 2020). Furthermore, the earlier meta-analytic study by Piquero et al. (2021) involving 18 empirical studies indicated increased domestic violence worldwide during the height of the COVID-19 lockdown. Demographic resilience is thus crucial to understand whether COVID-19-induced anxiety and stress have caused a breakdown in kinship relations (e.g., social capital) among the vulnerable artisanal fishing households.

The aforementioned three focus points of this case study are seldom addressed comprehensively in the scientific literature, but they are essential in building community resilience and sustainability and promoting human well-being in fishing communities (e.g., Folke, 2016; Matson, 2016). The idea is to use and support local knowledge and community-led action to enhance local capacity to persist in the face of change or crisis and to continue to develop with the ever-changing environments (Elsner et al., 2018; Binz and Truffer, 2017).

## Review of related literature

Building resilient and sustainable communities is a co-evolutionary process involving changes in diverse elements, with multiple and interdependent development pathways. During crisis transitions, various actors manage socio-ecological systems and form diverse governance structures, from local to global, that affect well-being, resources, capabilities, beliefs, strategies, and interests (Ostrom, 2009). Change and development in the system involve many kinds of agency or effort, such as sense-making, strategic calculation, learning, conflict resolution, power struggles, creating alliances, and making investments. Hence, crisis adaptation and building human well-being and community resilience are highly complex processes, and no single theory or discipline can comprehensively address issues and problems (Harley and Clark, 2020; Avelino, 2017; Geels, 2004). This is especially the case in the Philippines, where poverty is a widespread and persistent contextual concern, affecting the overall well-being of the populace. Inequality in the country also remains high, with the top 1% of Filipino earners contributing 17% of the national income. In comparison, only 14% come from the bottom 50% (Belghith et al., 2022).

In the Philippines, poverty incidence is defined as the proportion of Filipinos whose per capita income cannot sufficiently meet the individual’s basic food and non-food needs. The subsistence incidence benchmark is also used alongside the poverty incidence indicator. It is defined as the proportion of Filipinos whose income is insufficient to meet their basic food needs. The Philippines Statistics Authority (PSA) produces these official poverty estimates, derived using income welfare aggregates evaluated against per capita poverty lines that are set broadly following the cost of basic needs (CBN) approach (see PSA, 2022). The country’s average “poverty” and “subsistence” thresholds per month for a family of five were PHP12,030 (USD232.7, based on the 2020 exchange rate of USD1 = PHP51.7) and PHP8,379 (USD162.1), respectively (PSA, 2022). This means that on per capita per day basis, an individual Filipino needed approximately USD 1.6 to survive. This figure is comparatively lower than what was set in the earlier international poverty line of USD1.9 per capita per day for low-income countries as defined by the World Bank, which was recently updated to USD2.15 in September 2022 (World Bank, 2022b).

In 2021, the population poverty incidence in the Philippines was 18.1%, equivalent to around 19.9 million Filipinos living in extreme poverty. The poverty incidence in the Philippines was double the global benchmark of 9.1–9.4 % in 2021. Similarly, the proportion of food-poor Filipinos living below the USD1.0 subsistence threshold per capita per day increased to 1.04 million (5.9%) in 2021 from its 840,000 (5.2%) baselines in 2018. While these estimates of poverty thresholds received various criticisms because of their economic impracticality (Palatino, 2022), it is apparent that the poverty level in the Philippines has worsened due primarily to the COVID pandemic.

In addition to the official poverty estimates produced by PSA, other pertinent poverty indicators in the Philippines are the self-rated poverty (SRP) and self-rated food poverty (SRFP) collected quarterly nationwide by the Social Weather Stations (SWS) (see SWS, 2021). SWS is an independent social research institution in the country. SRP and SRFP can be construed as subjective well-being indicators based on people’s qualitative viewpoints. In 2019 pre-COVID, the nationwide average SRP and SRFP incidences were 45% and 32%, respectively. Although the countrywide SRP and SRFP averages did not widely differ pre-COVID in 2019 compared to peri-COVID situations in 2020 and 2021, these values wildly fluctuated quarterly across regions. For example, the Visayas region recorded a 70% SRP in the 2^nd^ quarter of 2021 against the national SRP value of 48%.

The Philippine response to COVID-19 has been described as draconian, militarised, and considered one of the longest and strictest lockdowns in the world (Hapal, 2021). Many provinces and cities were put into various categories of community quarantine starting in mid-March 2020, implementing numerous restrictive protocols, including limited mobility, wearing masks and face shields, and social distancing. Violations of these restrictions were met with heavy punitive action, marked with the heightened presence of uniformed personnel to enforce community quarantine protocols. The Philippines generally adopted two main types of community quarantines and their laxer modifications—the enhanced community quarantine (ECQ) and the general community quarantine (GCQ) (see IATF, 2020). The ECQ is equivalent to a complete lockdown regarding security. Under ECQ, all households are required to observe strict home quarantine. School and university classes were also suspended and moved to online mode. Mass gatherings were prohibited, government offices were run with a skeletal workforce, businesses were closed except for those providing essential goods and services, and mass transportation was restricted. In GCQ localities, public transportation was allowed at a reduced capacity, and select businesses could operate at 50 to 100% of their regular capacity, depending on their industry.

The Bayanihan to Heal as One Act (Republic Act or R.A. No. 11469) is a Philippines law enacted in March 2020 to combat the COVID-19 pandemic. Through RA 11469, the national government supported the most vulnerable communities, including artisanal fishers, through various social protection measures, zero-interest loans, and credit and cash-aid packages (Ferrer et al., 2021). In Aklan, while most received cash relief from the government, this arrived late (middle to end of 2020) and was often considered insufficient to cover the income lost due to the quarantine restrictions. Similar to the earlier report of Ferrer et al. (2021), the respective LGUs in the province of Aklan played a crucial role in supporting marginalised coastal households through food relief (e.g., rice and canned goods). Aklan’s local industries were also instrumental in building economic growth and resiliency. Micro, small, and medium enterprises (MSMEs) continued to produce various products (e.g., woven piña and abaca cloth, gifts, and housewares, processed meat and bakery products, native delicacies, and souvenir items), provide employment and ease the sufferings when COVID-19 struck (Villanueva, 2022). In addition, countless civil society initiatives and volunteers assisted stricken communities in Aklan and elsewhere (Maghanoy, 2021).

## Methods

### Study area

**Fig. 1:**
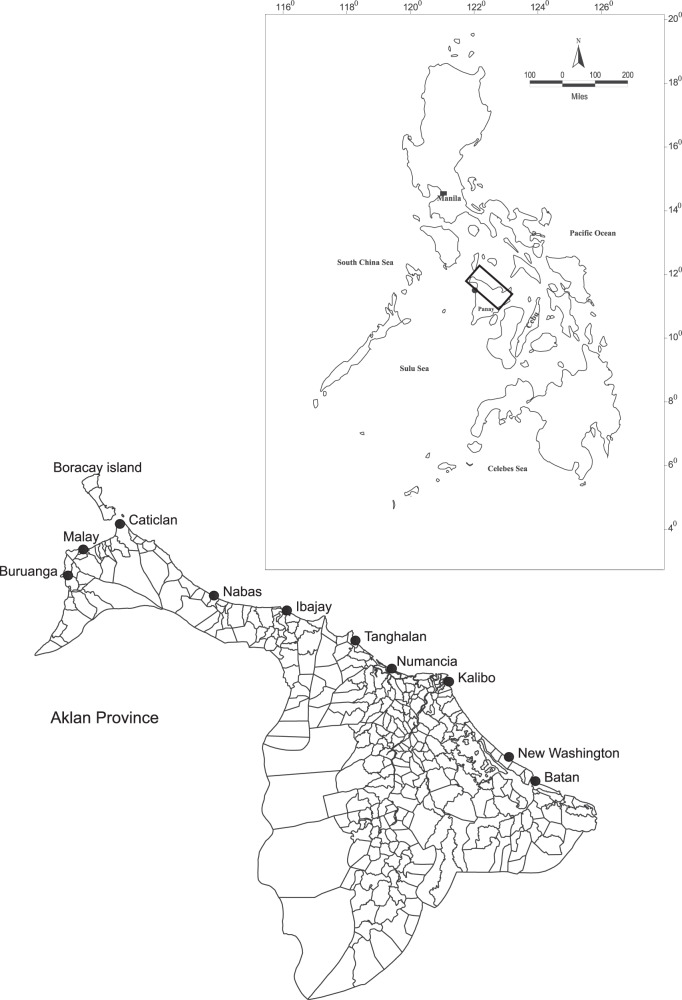
Sampling sites in the Province of Aklan, Philippines. All municipalities included in the survey are coastal.

The province of Aklan, located in the northwest portion of Panay Island (Fig. 1) in Western Visayas, Central Philippines, is well-known worldwide. First, it is the home of Boracay, a world-class beach island; second, it hosts the *Ati-Atihan*, a globally known annual religious festival. Eleven of the 17 municipalities in Aklan straddle the coastal area facing the Sibuyan Sea, considered among the most productive but highly overexploited seas in the Philippines. Coastal fisherfolk in the province are considered among the poorest of the poor, facing the economic consequences of severe fishery resource depletion and limited opportunities for alternative livelihoods (Maliao, 2019).

### Data collection and analyses

Despite the challenges posed by the spreading pandemic and community quarantine that commenced in March 2020, the research team proceeded with the household survey (May–December 2020) since Aklan was only under the less restrictive General Community Quarantine (GCQ) during the survey. All our field enumerators strictly adhered to the Philippine COVID-19 health protocols. It was decided that once one confirmed infection in the barangay (village) was detected, the survey would skip such a site.

Four hundred (400) respondents from the 10 coastal municipalities in Aklan were interviewed using a pre-tested semi-structured questionnaire implemented through face-to-face interviews. The respondents were representatives of distinct artisanal fishing households based on the most current local registry of fishers, selected through multistage stratified random sampling. Under the Philippines Fisheries Code (Republic Act 8550), fishers must register with the local municipal government. The registry is the basis for identifying local fishers who can fish within municipal waters. Therefore, each household in the survey had at least one family member engaged in municipal capture fisheries as a livelihood. The survey instrument covered various contextual attributes of each respondent representing demographic, social, occupational, and economic factors. The survey also covered the engagement of children in the household in fishing livelihood and their learning performance under the blended online learning modality. The survey addressed pre-(before) and peri- (during) COVID scenarios. The respondents’ perceived happiness index (used as a proxy indicator of subjective well-being) was also gauged using a 10-point scale where ten is the highest in 3 time periods (pre-COVID, peri-COVID, and post-COVID). The happiness indexes across the three time periods were compared using the Friedman test. The posteriori test followed the built-in pairwise comparison of the Freidman Test in SPSS (ver. 26), based on Dunn’s (1964) approach with Bonferroni correction.

### Limitations of the study

Our study focused on the impacts of COVID-19 on the economic and psychosocial well-being of coastal households with fishing as a primary source of livelihood. Our data were based mainly on household heads’ (e.g., parents’) livelihood engagements and perceptions. However, other household members generally engage in multiple livelihood strategies outside fishing. In the case of coastal communities in Aklan, most non-fishing livelihood engagements were intertwined with the marine tourism-based hospitality industry. Our survey and analyses did not address and disaggregate the direct impact of tourism-associated economic loss incurred by other family members of the household. Moreover, fishing households with larger family sizes would be disproportionately affected by the subsequent loss of income opportunities. Hence, there is a need for future studies to address the relative importance of other livelihood engagements (e.g., marine tourism) between and across different demographic factors (e.g., family size) within fishing communities so as to craft more locally relevant interventions during pandemics.

## Results

### Household contextual attributes

Four hundred (400) respondents from 400 distinct artisanal fishing households across the 10 sampling municipalities in the province of Aklan were interviewed. Males and females represented 63% (252) and 37% (148) of the total respondents. The average age of the respondents was 47.6 years old, ranging from 21–79 years old. The following were the distribution of respondent types: 1) father (59%), 2) mother (33%), 3) son (4%), 4) daughter (2%), and 5) relatives (2%). The relatives were composed mainly of parents of either the husband or wife in the household, indicating the general extended nature of Filipino families. Eighty-one per cent (81%) of our respondents were married; the rest were single, widowed, separated, or on live-in status. The average family size of the surveyed households was 5.4 members (2–15 family size). Fifty-nine per cent (59%) of the households had a family size of five or fewer (2–5 members), while the remaining 41% had a family size of more than five (6–15 members).

In addition to capture fisheries as the primary source of livelihood, most respondents supplemented household income with peripheral fisheries-related engagements, such as marketing, post-harvest, and aquaculture. The capture fisheries in the study sites employed various fishing methods, including cast nets, push nets, gill nets, ring nets, squid jigs, and pots. They were categorically artisanal because they were small-scale, low-capital, and mostly subsistence fishing. Many respondents were also engaged in fisheries marketing, directly selling their fishery products to serve as merchant intermediaries for other fishers. The post-harvest activities include deboning, drying, smoking, and salting fishery products. A small portion of the households was also engaged in aquaculture, primarily pond and cage farming. Most of the households owned their fisheries-related livelihood activities (86%), while the remaining 14% either rented or worked in the livelihood of their relatives.

Our respondents supplemented fisheries-related livelihood activities with various economic engagements. Our respondents’ top income source before the COVID-19 pandemic was fisheries-related, supplemented with seasonal hired labour works (primarily in construction and resort-related), private enterprise (primarily through home-based variety store), government employment, and farming. Although fisheries-related work remained a significant source of income during the pandemic, engagement from hired labour dwindled while government employment and private enterprise operation increased.

### Impacts of the pandemic on the livelihood and well-being of the fishing communities

**Fig. 2:**
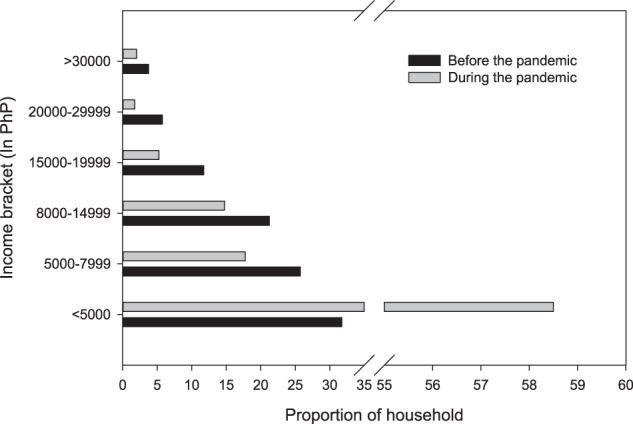
Income brackets of coastal fishing households pre-and peri-COVID. Income is derived from fishing-related livelihood only.

The COVID-19 pandemic severely disrupted the livelihood of artisanal fishing communities in Aklan, Philippines. Forty-three per cent (43 %) (*n* = 172) of the surveyed households reported unemployment of household member(s) due to being laid off primarily from tourism-related jobs. Twenty-six per cent (*n* = 105) of the households temporarily halted their fisheries-related livelihood due to lockdown restrictions implemented by the Philippine national and local governments. Ninety-five per cent of the households reported a decline in monthly income peri-COVID (Fig. 2). The monthly average pre-COVID income of PHP13,000 (USD254) dwindled by 31% to PHP9,000 (USD 176) peri-COVID. In particular, the proportion of households with a PHP5,000 (USD98) or less monthly income bracket increased from 32% pre-COVID to 59% peri-COVID. Conversely, 21% of the households with income above PHP15,000 (USD290) pre-COVID was reduced to 9% peri-COVID. Consequently, the proportion of households already living below the Philippine poverty threshold of PHP12,030 (USD232.7) monthly for a family of five members increased from 78% pre-COVID to 91% peri-COVID. Similarly, the proportion of food-poor households living below the subsistence threshold of PHP8,379 (USD162.1) for a family of five increased from 53% pre-COVID to 76% peri-COVID.

**Fig. 3:**
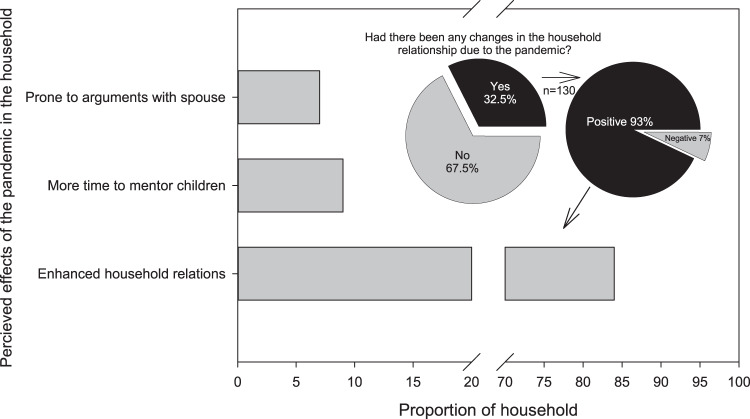
Perceived impacts of COVID-19 pandemic on household relationship dynamics. The positive impact of prolonged home quarantine period in family relations is attributed to the nurturing role of women in the household.

**Fig. 4:**
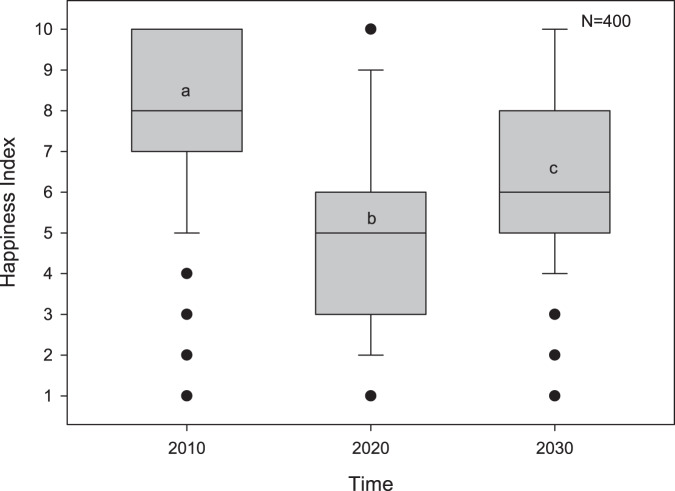
Boxplots of perceived happiness index between the three time periods in the context of the COVID-19 pandemic. Time treatments with dissimilar letters were significantly different according to Dunn’s post-hoc test after the Freidman test. The centerline of the box is the median. The bottom and top of the box are the 25th and 75th percentiles, and the whiskers below and above the box are the 10th and 90th percentiles. Points outside the whiskers are outliers.

Finally, the impact of the pandemic on the dynamics of household relationships was also assessed. Thirty-two per cent of the households (*n* = 130) reported changes, and 93% of this figure was positive, while the remaining perceived a negative impact (Fig. 3). On the other hand, the perceived happiness index was significantly different across the three time periods (pre, peri, and post-COVID) (Friedman test, *p* < 0.001, Fig. 4). The pairwise post-hoc Dunn test with Bonferroni adjustments showed that the perceived happiness index pre-COVID (MD = 8) was significantly higher compared to peri-COVID (Md = 5) and post-COVID (Md = 6) (*p* = 0.01). Similarly, the perceived post-COVID happiness index was significantly higher than peri-COVID (*p* = 0.001), which may imply hope of recovery.

### Engagement of children in fishing labour and its consequences for schooling

Seven hundred ninety-two (792) children from 400 artisanal fishing households were included in the analyses. Male and female children represented 53 and 47% of the sampling population. The average age of children engaged in fisheries-related livelihoods was 12, ranging from 5–17 years old. The proportion of children involved in labour pre-COVID was 15% (*n* = 119 children), while peri-COVID was 16% (*n* = 124 children). Of the children involved in labour peri-COVID, 35% (*n* = 43) and 46% (*n* = 57) have assisted their family’s livelihood with and without pay, respectively. Only 19% (*n* = 24) were in paid employment outside their immediate family livelihood. The most prominent fisheries-related livelihood engaged by 74% (*n* = 92) of the children was in capture fisheries, primarily in preparing, repairing, and maintaining fishing gear. The rest were engaged in other job clusters in aquaculture, post-harvest, and marketing. On average, the children received remuneration of less than PHP1,000 monthly (USD19.3). Children engaged in fisheries-related work primarily to gain experience and training (56%) and generate income for their family and personal needs (44%). Children generally engaged in fisheries-related work before or after school hours (42%) or during weekends (41%). Approximately 17% were involved in work during class hours.

**Fig. 5:**
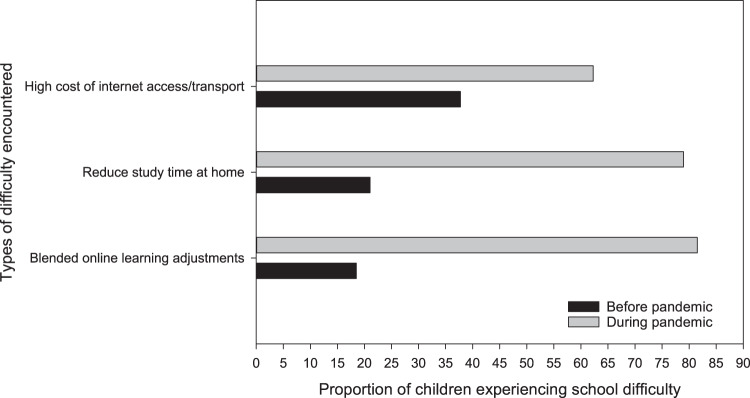
Types of learning challenges met by children under the blended learning modality. These learning difficulties peri-COVID is relative to the traditional face-to-face instruction delivery pre-COVID.

The proportion of households that reported their children encountered difficulties in their schooling increased from 15% pre-COVID to 57% peri-COVID (Fig. 5). This was mainly associated with the new blended learning modalities (online and modular), which increased learning difficulty by 81% peri-COVID compared to the 19% baseline difficulty pre-COVID during traditional face-to-face learning mode. Moreover, respondents also cited the high cost of schooling peri-COVID, primarily associated with the additional expenses incurred (i.e., buying data load for mobile devises) from accessing online classes. Finally, 37 (5%) of 792 children dropped out of school to gain employment or assist the family peri-COVID.

## Discussion

The COVID-19 pandemic disproportionately impacted the economic well-being of coastal fishing communities in Aklan, Philippines, primarily in two ways. First, the COVID-19 pandemic severely disrupted the marine tourism-based hospitality industry, with all tourism activities halted during government-implemented lockdowns in 2020 and 2021. Marine tourism is highly interwoven with the livelihoods of coastal fishing communities in Aklan province and in many parts of the Philippines. A case in point is Boracay Island, considered one of the Philippines’ top tourist destinations for foreign and local tourists, located in the northern part of the province. In 2019, Boracay Island was visited by 1.7 million tourists, half of which were international, generating nearly PHP 50 billion in tourism income (Abad, 2019). The tourism industry in Boracay Island alone attracts thousands of local employments through its various subsectors in food, transportation, lodging, and recreation. The financial cost of the Boracay lockdown in the first three months (March-June 2020) of the pandemic was estimated to be around PHP 12 billion (USD 232 million) (PNA, 2020). Hence, the complete cessation of all tourism activities nationwide during the pandemic triggered a chain reaction across all economic sectors. This tourism disruption reverberated in the coastal fishing communities in the province through job losses of family members and lost/reduced economic opportunities in the fisheries industry. This situation is manifested in our study, where 43% of the surveyed households had family members dismissed from tourism-related jobs during the lockdown. The disruption in the tourism industry was also reflected in the province exhibiting higher than the national average poverty incidence of 20.2% in 2021 (PSA, 2022).

Second, the COVID-19 pandemic lockdowns directly affected various facets of the fisheries value chain, including capture and production, marketing, and distribution (e.g., Manlosa et al., 2021; Robins et al., 2020). Such economic perturbations were debilitating for artisanal fishing communities with limited livelihood options (Maliao et al., 2009). These communities were already at the forefront of life-threatening impacts of climate change (Suh and Pomeroy, 2020). Furthermore, artisanal fishing communities have limited access to traditional financial services such as savings, credit, and insurance (Pomeroy et al., 2020), thus needing swift assistance during a crisis. But government aid in the Philippines during the pandemic was fragmented and delayed (Ferrer et al., 2021). More importantly, lockdowns were implemented in large areas for almost two years, thus worsening the adverse economic effects. Granular lockdown or a micro-level quarantine for areas identified as “critical zones” would have a less debilitating impact on local communities’ economic and psychosocial well-being (see Parrocha, 2021).

The province of Aklan was put under Enhance Community Quarantine (ECQ) from 23 March to 30 April 2020 and eased to General Community Quarantine (GCQ) for the rest of 2020 and 2021. While fishing was allowed during the quarantine periods (DA-BFAR, 2020), restrictions on mobility and fear of infection resulted in an overall reduction of fishing efforts to the total abandonment of fishing livelihood. This was coupled with general fishery product depreciation because of reduced demand due to the closure of restaurants and hotels and the complete shutdown of tourism activities. Similar COVID-19 aftermath trajectories are reported elsewhere (Hoque et al., 2021; Lau et al., 2021). Because of mobility restrictions, transportation costs have spiked because of the implemented social distancing in public vehicles, making fishery product marketing challenging and costly. Mirasol (2020) reported that the closure of ice plants and long queues at checkpoints associated with locational border control and mobility restrictions in many parts of the Philippines peri-COVID caused spoilage of perishable fishery products. In Aklan, such disruptions further burden women of the household as they are primarily responsible for marketing tasks. Indeed, women in the Philippines have been disproportionately affected by the pandemic-induced socio-economic perturbations because many operate home-based enterprises, such as small food stalls called “*carinderias*” or small convenience shops called “*sari-sari stores*”. These informal sectors of the economy, valued at PHP5.013 trillion (USD 98 billion) to GDP, are the first casualties during lockdown periods (Leyesa and Flores-Obanil, 2021). Ferrer et al. (2021) pointed out that a similar scenario occurred in past disasters where women disproportionately shouldered the burden of food insecurity. Most households coped by selling their fishery products at home and online through social media platforms (primarily Facebook) and mobile texting. Facebook is prominent in the Philippines, with 1 in every 5 Filipinos using the social media platform (SWS, 2019). While this alleviates the local marketing bottleneck, this also adds additional expenses on internet subscriptions.

The Philippine draconian impositions of various restrictions to stem the tide of COVID-19 risks resulted in the deterioration of the economic well-being of artisanal fishing communities in Aklan, as reflected in the depression of various economic indicators at the level of households. Foremost, unemployment based on the surveyed households has worsened, quadrupling the 10.4% national unemployment average of 2020. Rising local unemployment is further aggravated by the influx of community members recently laid off or furloughed from their work locally and abroad. Indeed, the Philippines logged the highest unemployment rate at 17.6% in April 2020 at the onset of the COVID-19 pandemic (Crismundo, 2022). Due to reduced economic activities and pervasive unemployment, the monthly average income of artisanal fishing communities engaged in fisheries-related livelihood in Aklan exhibited a steep decline. Consequently, the proportion of households living below the poverty line has ballooned by 13%, pushing 91% of the surveyed household to extreme poverty peri-COVID. This means that nine in every 10 artisanal fishing households in the coastal communities in Aklan were living below the national poverty threshold of approximately USD 1.6 per capita per day peri-COVID. This result is considerably higher than the 60% SRP value for the whole province of Aklan in the last quarter of 2020 based on the SWS survey (SWS, 2021). Similarly, the proportion of food-poor households increased by 23% peri-COVID, pushing the proportion of households living below the USD1.0 per capita per day subsistence threshold to 76%. This signifies that three in every four artisanal fishing households in Aklan could barely afford food peri-COVID. If extrapolated for the entire coastal households in the province of Aklan, this result means that 62,000 households (*N* = 82,025) or 305,558 individuals (*N* = 402,050) were too poor to be able to satisfy daily food requirements peri-COVID. Again, this result is considerably higher than the 39% SRFP for the whole province of Aklan in the last quarter of 2020 based on the SWS survey (SWS, 2021).

Compared to national and regional benchmarks, the artisanal fishing households in Aklan came out as among the poorest of the poor in the Philippines. Indeed, the poverty situation in coastal communities could even be worse than the official poverty incidence threshold indicates. Forty-one per cent (41%) of the surveyed households had larger family sizes^2^ of more than five (6–15 members), signifying that poverty was more adverse in larger households with limited income. The economic cost of the pandemic further worsened as expenditure in various categories increased peri-COVID (e.g., food, mobile gadgets and internet access, and healthcare). Therefore, the COVID-19 pandemic severely disrupted the economic resilience of artisanal fishing households in the coastal communities in Aklan. Ferrer et al. (2021) recommendations to enhance household resilience are relevant in the artisanal fishing communities in Aklan. These include 1) developing household social capital, 2) livelihood diversification, 3) financial inclusion through savings, credit, and insurance, and 4) retooling post-harvest handling and processing skillsets.

Another collateral damage of the COVID-19 pandemic is the increase in child labour in the Philippines (Calleja, 2020). Child labour was pervasive in the fishing and aquaculture sectors (Clark et al., 2019; Ratner et al., 2014) but has worsened during the COVID-19 pandemic (Kundu, 2020). For example, in the province of Aklan, the already impoverished situation pre-COVID pushed 16% of the 792 children from the 400 artisanal fishing households to engage in fisheries-related work. Although it is worth noting that the COVID-19 pandemic has not directly increased this figure (pre-COVID and peri-COVID child labour incidences exhibited minimal increase), the COVID-19 pandemic amplifies the already depressed situation of the artisanal fishing communities. The worsening poverty is reflected in the 5% of children who stopped schooling peri-COVID to augment their household income. This result again highlighted the economic vulnerability of artisanal fishing communities because they lacked alternative sources of income to cushion disruptions in their fishing livelihood. Hence, the increasing poverty due to reduced economic activity, unemployment, and lack of opportunities for job multiplicity peri-COVID pushed artisanal fishing communities to rely on immediate family members (including children) to augment the already limited household income. The issue of child labour due to the exacerbation of poverty associated with the COVID-19 economic perturbations was further complicated by school closures. As schools are closed during the pandemic affecting 1 billion learners in over 130 countries, there is mounting evidence that child labour is rising (ILO, 2020).

The Philippine government adopted blended learning modalities (online and printed modules) to replace the traditional face-to-face education delivery during school closures. This blended learning arrangement compelled by the COVID-19 pandemic reveals asymmetrical readiness between and among the learning providers and home-based mentors and learners alike. Most artisanal fishing households in the coastal communities of Aklan had limited access to the technology and limited support systems necessary for learning under the new blended online learning modality. Furthermore, blended learning often necessitates the involvement and supervision of parents and guardians, who are unaware or under-equipped for their responsibilities under the current educational reform (Lardizabal-Dad, 2020). Another challenge of home-based learning is the conduciveness of the learning environment at home and technological access and proficiency. The typically large family sizes, aggravated by the limited, mostly shared space characteristic of the housing in coastal communities, provide a less-than-ideal environment for learning, similar to an earlier report by Barrot et al. (2021). This issue explains why children under the survey reported reduced time invested in studying peri-COVID. Furthermore, the unequal capacity of access to gadgets and the Internet for online education resources further exacerbated learning inequalities (Agaton and Cueto, 2021). It is important to note that internet connectivity in the Philippines is among Asia’s slowest and most costly (Salac and Kim, 2016). For example, only 18% of the Philippines households have internet subscriptions (Santos, 2021b). With limited internet providers, internet penetration in rural areas such as the remote coastal communities in Aklan is often unreliable, even if some households have the financial capability for a subscription. Indeed, the prohibitive cost of internet subscriptions and internet access difficulties are among the identified challenges of blended online learning in Aklan.

On the academic side, most educational institutions in the Philippines are not prepared for blended online education (Joaquin et al., 2020; Toquero, 2020), particularly in designing effective and appropriate modular learning under the prevailing K-12 educational reform. To develop online and module instruction, teachers must consider several factors, including lesson structure, content presentation, need for collaboration and interaction, ensuring timely feedback, motivation of students, social relationships affecting student learning, and even mental health. Furthermore, income losses and school closures significantly impact students’ and teachers’ motivation and family engagement in education. These combined scenarios contributed to the escalating difficulties the children in Aklan encountered with blended learning. More importantly, blended learning reduced the amount of instruction with no social interplay with classmates, reducing the children’s human capital. Indeed, the world’s most extended school closure implemented in the Philippines is dubbed as an impending “learning and child development catastrophe” (Santos, 2021b). The National Economic Development Authority (NEDA) estimated that prolonged school closures could result in an estimated productivity loss in the Philippines, equivalent to $219 billion over the next 40 years (Santos, 2021b).

Finally, the combination of paranoia in getting COVID-19 illness, the social stigma of being infected, rising unemployment and decreased income, reduced economic activity, and increasing poverty heightened psychosocial distress among the artisanal fishing households in Aklan. The new challenges of blended learning further worsened these issues. This worsening sense of psychosocial well-being is reflected in the significant decline in their happiness index peri-COVID, similar to the national (SWS, 2021) and worldwide trends (Zacher and Rudolph, 2021; Wang et al., 2020). However, the marked decline of the happiness index in coastal communities in Aklan did not result in animosity within the household (e.g., Piquero et al., 2021; Boserup et al., 2020). This apparent tenacity reveals the resiliency of Filipino culture against such upheavals, even for vulnerable and impoverished artisanal fishing communities. Over a quarter of our respondents perceived the prolonged home lockdown as enhancing household interpersonal relations. This can be attributed to the role of women (i.e., mothers, grandmothers, sisters) as the “*ilaw nang tahanan*” (light of the home), providing unity, stability, and direction to households and communities during times of crisis. In general, women were observed to organise communities and promote “*bayanihan*” or self-help measures of sharing, working, and surviving together. Women assisted in the construction of community food pantries, provided information and assistance to families seeking government assistance, prevented age-and gender-based violence in the home, cared for the sick and elderly, and implemented other socio-economic survival strategies (Ofreneo, 2020). In Aklan, the small proportion that reported that lockdowns increased household tension was generally attributed to a constrained family budget and the difficulty of assisting children with blended learning. It is worth noting that the mothers and women in the family predominantly serve as home mentors of children in Aklan. Hence, women in the households are disproportionately affected because they perform multiple roles, ranging from fisheries-related responsibilities to obligations along the gradient of maternalism and family member nurturing and mentoring (Maliao and Polohan, 2008). Furthermore, the resiliency of the artisanal fishing households in Aklan against the pandemic-induced perturbations can also be traced to their strong religious background and “*bahala na*” (come what may) coping mechanisms. The expression “*bahala na*” (originating from ‘*Bathala*,’ signifying God or Divine) suggests a mixed attitude of optimistic acceptance or fatalistic resignation during challenging situations to divine providence (e.g., Rosales and Arabit, 2021). The expression also has an aspect of “responsible deliberative act of choosing” pathways in life (Gripaldo, 2005).

## Conclusion

This study demonstrates the cascading, multifaceted, and debilitating impacts of the COVID-19 pandemic on the socio-economic and demographic resilience of the artisanal fishing communities in Aklan, Philippines. The crisis hit while they were already facing the challenges of declining catch, resource degradation, and climate change. The socio-economic and demographic disruptions brought by the COVID-19 pandemic were more pronounced in these vulnerable communities because they lacked livelihood multiplicity options, inadequate social security, and limited access to traditional financial services. Furthermore, the socio-economic shock brought by the draconian response of the Philippine government to the COVID-19 pandemic further revealed the limitations and risks of a tourism-dependent economy in which coastal fishing communities become tightly interwoven. This is highlighted in the artisanal fishing communities in Aklan, Philippines, with nine in every 10 households driven to extreme poverty peri-COVID. This economic hardship was more pronounced in larger households, underscoring the importance of mainstreaming reproductive health and women’s education and economic empowerment in coastal communities. The issue of responsible family planning within coastal communities becomes more relevant in the context of promoting household resilience amidst declining resources and crises. There was also a real risk of exacerbating child labour within artisanal fishing communities amidst the need to reduce costs and boost earnings. The COVID-19 pandemic also ushered in new challenges for parents, guardians, and learners under the abrupt educational reform. The prolonged school closures in the Philippines, even alleviated with the blended online learning modality, exposed children’s human capital to further erosion. The responses to address the educational needs of children could focus on providing assistive technologies to maintain learning from home and increasing internet coverage and bandwidth. The international and national collaboration between universities/schools should also be emphasised to deploy and share quality learning resources for local communities. Consequently, parents need more ICT training, pedagogical support, and psychological guidelines on motivating children under the new educational reform. Finally, there should be more investigation on how to make the best of television and radio as learning mediums for those who do not have internet access or those with disabilities. Radio and television are widely available in rural areas in the Philippines.

Finally, this study has shown that transitions towards community resilience or sustainability in a crisis (like the COVID-19 pandemic) are complex and not linear. Building human well-being, community resilience, and sustainability is highly context-dependent and involves multiple actors, processes, and interactions. In the case of Aklan, there is evidence that government agencies, business groups, and civil society organisations were all actively involved in addressing various issues and concerns on the health, nutrition, education, and general well-being of affected communities. Efforts, however, were not well coordinated, and consultative planning and implementation with local communities were lacking. Local and national governmental agencies must provide an enabling environment to provide targeted support, including resources (i.e., manufactured and knowledge capital) and the creation of granular lockdowns to enable areas with low infection rates to continue with their livelihood and social activities. Nationally supported and locally implemented guidelines and responses to address well-being concerns must be framed within a “whole-of-society” approach. Notwithstanding governance challenges and limitations, the COVID-19 pandemic illustrates the high social capital in the study sites, exemplified by women’s stabilising and nurturing role. This signifies cooperative and nurturing actor relationships can be generated even in crises. Hence, more should be done to better situate and facilitate women’s opportunities and empowerment in times of crisis. That way, women and other local actors can help build a better and more resilient “normal” (see U.N. Philippines, 2021).

## Data Availability

The datasets generated and analysed during the current study are available from the corresponding author upon reasonable request.

## Footnotes

1 Human well-being is a broad and multilevel concept associated with being healthy (e.g., sound and functional), happy and prosperous (e.g., enjoy a satisfactory quality of life), and more recently considered analogous with income and consumption levels (see Breslow et al., 2016; McGillivray and Clarke, 2008). Human well-being is supported by stocks of capital on which people draw and will draw on in the future to subsist and to improve their lives. These capital include natural (e.g., environment, biodiversity, etc.), social (e.g., econo-politico-cultural arrangements governing interactions), human (e.g., distribution, health, education, etc.), manufactured (e.g., industrial systems, cities, telecom, energy, etc.), and knowledge capital (e.g., scientific findings, technology, skills, expertise, etc.) (see Matson et al., 2016; Breslow et al., 2016).

2 Wealthiest nations with the greatest food security have the lowest fertility rates, whereas the most food-insecure countries have the highest fertility rates. Thus, food-insecure households tend to have bigger numbers. A possible solution to overpopulation is not to force people to have fewer children. It is to raise the poorest nations out of poverty through democratic governance, free trade, access to birth control, and the education and economic empowerment of women (see Bailey, 2015).
